# ECG Marker of Adverse Electrical Remodeling Post-Myocardial Infarction Predicts Outcomes in MADIT II Study

**DOI:** 10.1371/journal.pone.0051812

**Published:** 2012-12-14

**Authors:** Larisa G. Tereshchenko, Scott McNitt, Lichy Han, Ronald D. Berger, Wojciech Zareba

**Affiliations:** 1 The Division of Cardiology, Department of Medicine, Johns Hopkins University School of Medicine, Baltimore, Maryland, United States of America; 2 The Division of Cardiology, University of Rochester Medical Center, Rochester, New York, United States of America; 3 Whiting School of Engineering, Johns Hopkins University, Baltimore, Maryland, United States of America; University of Minnesota, United States of America

## Abstract

**Background:**

Post-myocardial infarction (MI) structural remodeling is characterized by left ventricular dilatation, fibrosis, and hypertrophy of the non-infarcted myocardium.

**Objective:**

The goal of our study was to quantify post-MI electrical remodeling by measuring the sum absolute QRST integral (SAI QRST). We hypothesized that adverse electrical remodeling predicts outcomes in MADIT II study participants.

**Methods:**

Baseline orthogonal ECGs of 750 MADIT II study participants (448 [59.7%] ICD arm) were analyzed. SAI QRST was measured as the arithmetic sum of absolute QRST integrals over all three orthogonal ECG leads. The primary endpoint was defined as sudden cardiac death (SCD) or sustained ventricular tachycardia (VT)/ventricular fibrillation (VF) with appropriate ICD therapies. All-cause mortality served as a secondary endpoint.

**Results:**

Adverse electrical remodeling in post-MI patients was characterized by wide QRS, increased magnitudes of spatial QRS and T vectors, J-point deviation, and QTc prolongation. In multivariable Cox regression analysis after adjustment for age, QRS duration, atrial fibrillation, New York Heart Association heart failure class and blood urea nitrogen, SAI QRST predicted SCD/VT/VF (HR 1.33 per 100 mV*ms (95%CI 1.11–1.59); P = 0.002), and all-cause death (HR 1.27 per 100 mV*ms (95%CI 1.03–1.55), P = 0.022) in both arms. No interaction with therapy arm and bundle branch block (BBB) status was found.

**Conclusions:**

In MADIT II patients, increased SAI QRST is associated with increased risk of sustained VT/VF with appropriate ICD therapies and all-cause death in both ICD and in conventional medical therapy arms, and in patients with and without BBB. Further studies of SAI QRST are warranted.

## Introduction

Electrical and structural remodeling is a continuous process that begins early after myocardial infarction (MI), and ventricular arrhythmias occur late in the remodeling manifestation [1], [2]. Post-infarction structural remodeling is well described [3] and is characterized by scar formation [4] in the central and border zone areas [5], progressive dilatation and distortion of cavity shape, progressive vasculopathy due to activation of the neurohumoral pathways, left ventricular (LV) dysfunction, fibrosis, and hypertrophy of non-infarcted myocardium [6], [7].

ECG signs of post-MI scar (deep and broad Q wave, QS waves, fragmented QRS, bundle branch block [BBB], fascicular block) are well known. It is known that post-MI scar ECG features can change over time, e.g. Q waves may resolve within one year post-MI [8]. However, complete ECG manifestations of post-MI electrical remodeling are not entirely understood. LV hypertrophy is recognized as a strong risk factor of ventricular arrhythmias [9], [10]. ECG marker of LV hypertrophy, Cornell voltage-duration product, was shown to be associated with the risk of sudden cardiac death (SCD) [11]–[13]. In hypertrophic cardiomyopathy patients, large sum of the 12-lead QRS-amplitudes predicted sudden cardiac arrest [14]. Adverse post-MI remodeling is characterized [15] by the progressive apoptotic loss of cardiomyocytes in areas remote from the scar location, insufficient pathological hypertrophy and thereby ventricular wall thinning and fibrosis, and progressive LV dilatation [6]. More favorable post-MI remodeling is characterized by an inhibition of post-MI fibrosis, removal of excess collagen, and compensatory hypertrophy with adequate angiogenesis in the remote myocardium [16]. However, distinct ECG characteristics of adverse post-MI remodeling, in comparison to more favorable post-MI remodeling have not been systematically described. Moreover, presence of bundle branch block (BBB) on ECG significantly limits interpretation and prognostic value of ECG, and to date, no reliable ECG marker of electrical remodeling in BBB has been developed.

Electrical remodeling involves both depolarization and repolarization phases of the cardiac cycle, which should be taken into account in order to quantify post-MI electrical remodeling. Recently we developed a novel ECG marker, associated with the risk of ventricular arrhythmias in primary prevention ICD patients [17], [18]. We hypothesized that our new marker, sum absolute QRST integral (SAI QRST), as a marker of adverse electrical remodeling, predicts SCD and sustained ventricular tachycardia (VT)/ventricular fibrillation (VF) with appropriate ICD therapies in post-MI patients with systolic dysfunction, participants of the landmark MADIT II ICD trial, in both arms (ICD and conventional medical therapy), both in patients with and without BBB.

## Methods

### MADIT II Study Population

The post hoc analysis presented in this manuscript was performed using prospectively collected data of the MADIT II trial. Design and results of the MADIT II study are well-described [19], [20]. Baseline 10-minute high resolution (sampling rate 1000 Hz and amplitude resolution 0.1526 µV) orthogonal ECGs were recorded at rest via digital Spacelab-Burdick 6632 Holter recorders (Spacelab-Burdick, Milton, WI) [19], [21]. We analyzed data of participants with available 10-minute ECGs in both the ICD arm and in the conventional medical therapy arm. The original MADIT II trial 4-year (median 20 months) period of follow-up was included in our analysis. Combined endpoint, defined as either SCD or sustained VT/VF with appropriate ICD therapies served as the primary end point in our study. Total mortality served as a secondary end-point.

### Sum Absolute QRST Integral Measurement

Baseline ECG analysis was performed by investigators fully blinded to the study outcomes and patients clinical characteristics (L.H., L.G.T.). Customized MATLAB (MathWorks, Inc., Natick, MA) software application for data analysis was developed in Tereshchenko’s laboratory. Noisy recordings were excluded from analysis. ECG recordings in atrial flutter and atrial fibrillation with large visible f-waves were excluded, whereas those with atrial fibrillation and small f-waves were analyzed if f-waves did not preclude precise Tend detection. ECG recordings with conduction abnormalities (AV block, BBB) were analyzed. Premature ventricular contraction (PVC) beats and one consecutive sinus beat after PVC were excluded. ECG recordings with frequent PVCs (bigeminy, trigeminy), as well as ventricular-paced rhythm, were excluded. Fiducial points [beginning of Q or R wave and end of T wave] were detected automatically on XYZ leads by previously described methods [22], [23]. The accuracy of the automatically detected fiducial points on each beat was visually checked by investigators (L.H., L.G.T.). The “zero” value of the baseline was determined by the position of the T end [23]. Trapezoidal Riemann sums (1 ms interval size) were used to calculate the area under the QRST curve. Absolute QRST integral was measured as the arithmetic sum of areas under the QRST curve, averaged during a 3-minute epoch. The sum magnitude of three orthogonal leads absolute QRST integral (SAI QRST) was calculated according to the following equation:
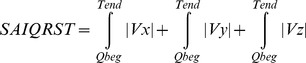



Magnitude of spatial QRS vector and magnitude of spatial T vectors were measured as previously described [24]. J-point deviation was measured as interloop distance metric the shortest distance between QRS and T loops as previously described [25].

### Statistical Analysis

Statistical analysis was performed in the Statistical Core Laboratory of the Heart Research Follow-up Program at the University of Rochester. Normally distributed continuous variables were compared using the independent samples *t* test, and results are presented as mean±standard deviation (SD). Categorical variables were compared using Pearson’s chi-square test. SAI QRST distribution was categorized by quartiles. SAI QRST was regressed on QRS width, mean magnitude of spatial QRS and T vectors, 3D J-point deviation amplitude (interloop distance) and QT duration. These ECG metrics in patients with SAI QRST in the highest quartile and in 3 lower quartiles were compared by *t*-test. Kaplan-Meier survival analysis was used to test the predictive value of SAI QRST quartiles for SCD/VT/VF and all-cause mortality. The univariate Cox regression analysis was performed with SAI QRST as a continuous variable, and SAI QRST dichotomized at the 75^th^ percentile. Multivariable Cox regression analyses were performed to adjust for clinical and demographic variables associated with endpoints in the MADIT II study as shown previously (age >70 years, blood urea nitrogen (BUN) >25 mg/dl, New York Heart Association (NYHA) heart failure (HF) class >II, QRS >120 ms, BBB status). Interaction between SAI QRST and BBB status, as well as between SAI QRST and study arm (ICD vs. conventional therapy) was tested in Cox regression models for SCD/VT/VF and all-cause mortality. Treatment-by-interaction term analysis was employed to evaluate the benefit of the ICD in patients with the highest quartile of SAI QRST and in those within the 3 lower quartiles of SAI QRST. A *P*-value of <0.05 was considered significant. SAS 9.2 (SAS Institute Inc, Cary, NC) was used for data analysis.

## Results

### Study Population

Only patients with available baseline orthogonal ECGs were included in this analysis. Baseline orthogonal ECGs of 796 MADIT II participants were reviewed. We excluded 32 ECG recordings due to noise, 7 ECGs due to coarse atrial fibrillation, 4 ECGs due to frequent PVCs, 3 ECGs due to ventricular pacing, and the remaining 750 ECGs were analyzed. Out of 750 analyzed patients, 448 (59.7%) had an ICD implanted and 302 (40.3%) received conventional medical therapy. Right BBB was present in 46(6.1%) patients, left BBB in 123(16.4%) patients and atrial fibrillation in 7 (9.3%) patients. During follow-up, 104 patients (23%) experienced appropriate ICD therapies, 48 patients (6.4%) had SCD, and 26 patients (3.5%) had non-sudden cardiac death.

### ECG Characteristics of Adverse Electrical Remodeling and SAI QTST

**Figure 1:**
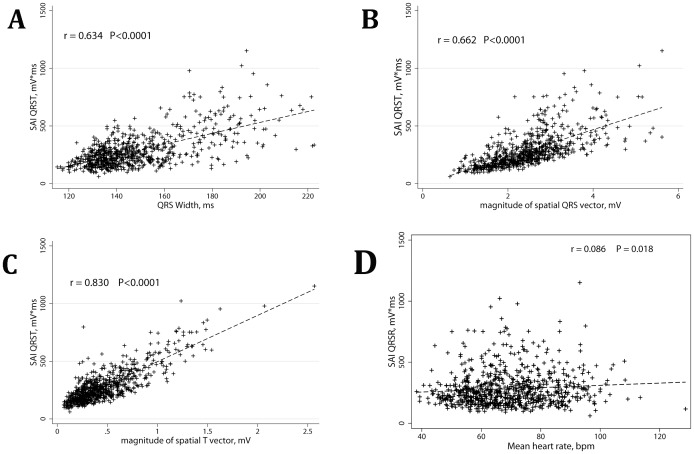
Correlations between QRS width and SAI QRST (A), between mean magnitude of spatial QRS vector and SAI QRST (B), between mean magnitude of spatial T vector and SAI QRST (C), and between mean heart rate and SAI QRST (D). Linear regression fitted line is shown in each graph.

Patients in the highest quartile of SAI QRST (>334 mV*ms) had significantly wider QRS with predominantly left BBB pattern; they were older and had a higher occurrence of elevated BUN level (Table 1). Wide QRS, prolonged QTc, significantly larger magnitudes of spatial QRS and T vectors, and twice larger amplitude of J-point deviation characterized ECG pattern of adverse electrical remodeling in patients with SAI QRST in the highest quartile (Table 2). SAI QRST strongly correlated with QRS width (r = 0.634; p<0.0001; Figure 1A), mean magnitude of spatial QRS vector (r = 0.662; p<0.0001; Figure 1B) and mean magnitude of spatial T vector (r = 0.830; p<0.0001; Figure 1C). All together depolarization characteristics (mean QRS duration and mean magnitude of spatial QRS vector) accounted for three quarters of the variance in SAI QRST (R^2^ = 0.75; p<0.0001). Repolarization characteristics collectively (mean QT interval, J-point deviation amplitude, measured as interloop distance, mean magnitude of spatial T vector) accounted for approximately three quarters of the variance in SAI QRST (R^2^ = 0.74; p<0.0001) as well. In bivariate linear regression analysis, mean magnitude of spatial T vector accounted for 69% of the variance in SAI QRST value (R^2^ = 0.69; p<0.0001), whereas mean magnitude of spatial QRS vector accounted for only 44% of the variance in SAI QRST value (R^2^ = 0.44; p<0.0001). In contrast to magnitudes, impact of intervals on SAI QRST in bivariate linear regression analysis was smaller and varied from 40% for QRS width (R^2^ = 0.40; p<0.0001) to 2% for QT interval (R^2^ = 0.02; p<0.0001). Interestingly, in bivariate regression analysis, 25% of SAI QRST variance was due to J-point deviation amplitude (R^2^ = 0.25; p<0.0001). No meaningful correlation between mean heart rate and SAI QRST was observed (Figure 1D).

**Table 1:** Baseline clinical characteristics of patients with SAI QRST, dichotomized at the 75^th^ percentile (>334 mV*ms).

	SAI QRST Q1-Q3 (n = 562)		SAI QRST Q4 (n = 188)		P
	Missing, n	%	Missing, n	%	
Age ≥65 y	0	41.0		57.8	**<0.001**
Female	0	16.9	0	21.4	0.164
NYHA class II-IV	7	61.2	2	68.1	0.090
EF <25%	0	41.6	0	56.7	**<0.001**
BMI ≥30	0	36.1	0	23.0	**0.001**
QRS >120 ms	4	15.4	3	68.5	**<0.001**
LBBB	7	3.6	4	55.7	**<0.001**
RBBB	7	6.7	4	4.9	0.399
HR> = 80 bpm	8	24.5	3	31.0	0.083
CABG	0	52.6	0	61.0	**0.046**
non-CABG revasc	8	47.2	1	34.4	**0.002**
MI <18 months	37	70.3	7	77.8	0.055
BUN >25 mg/dL	5	22.6	1	29.6	0.054
Creatinine ≥1.4 (Q4)	2	20.1	0	26.2	0.081
Diabetes mellitus	1	34.7	0	30.5	0.291
Treatment (ICD arm)	0	61.1	0	55.6	0.185
Inducible VT narrow defin	294	34.6	99	34.1	0.934
Beta-blockers	0	68.4	0	54.0	**0.0001**
Calcium ch block	0	12.3	0	19.3	**0.017**
ACE-I	0	79.2	0	77.5	0.627
ARBs	0	11.5	0	16.6	0.074
Amiodarone	0	6.0	0	5.3	0.727
Class I AA	0	2.5	0	5.3	0.110
Diuretics	0	69.4	0	80.2	**0.005**
Digitalis	0	54.7	0	62.0	0.080

**Table 2:** Outcomes and baseline ECG parameters in patients with SAI QRST, dichotomized at the 75^th^ percentile (>334 mV*ms).

	SAI QRST Q1–Q3 (n = 562)			SAI QRST Q4 (n = 188)		P
	Missing, n		Mean, %	Missing, n	Mean, %	
ICD therapy for VT/VF	229		20.1	85	36.3	0.001
All-cause death	0		11.4	0	19.3	0.006
CHF hospitalization	2		15.9	2	22.2	0.050
CHF hosp or death	2		21.9	2	31.9	0.006
ReMI hospitalization	2		3.4	1	3.2	0.916
Cardiac death	2		8.9	1	17.7	0.001
SCD	5		5.7	7	8.9	0.136
Non-sudden cardiac death	5		2.7	7	6.1	0.030
QTc ±SD, ms	467±35			492±41		<0.0001
Mean heart rate±SD, bpm	68.1±13.9			71.8±13.4		0.001
QRS duration±SD, ms	114.4±13.8			166.2±22.5		<0.0001
Spatial QRS vector magnitude±SD, mV		2.19±0.65		3.19±0.82		<0.0001
Spatial T vector magnitude±SD, mV		0.35±0.17		0.81±0.36		<0.0001
Interloop distance±SD, mV	0.025±0.026			0.069±0.070		<0.0001

### SAI QRST and Clinical Characteristics of Patients

Patients with the SAI QRST in the highest quartile were older, had lower body mass index (BMI), wider QRS and more likely had left BBB (Table 1). Interestingly, they were more likely to have a history of revascularization procedures and of recent MI (<18 months ago) and were more often on calcium channels antagonists and diuretics, but were less often on beta-blockers.

During study follow-up patients with the highest quartile of SAI QRST had about twice the rate of appropriate ICD therapies due to sustained VT/VF, and at the same time about twice the rate of non-sudden cardiac death and hospitalizations due to heart failure exacerbations, as compared to patients with SAI QRST in 3 lower quartiles (Table 2).

### Time after Myocardial Infarction and SAI QRST

**Figure 2:**
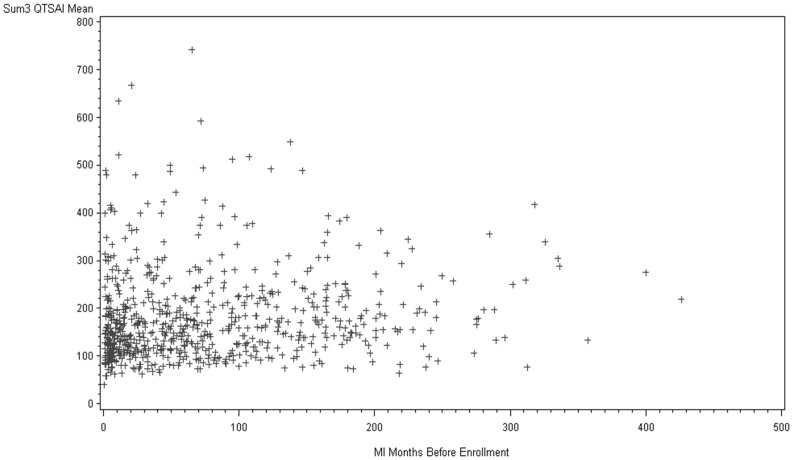
Correlation between the time after myocardial infarction and SAI QRST. P = 0.003.

SAI QRST correlated with time after MI (Figure 2). Statistically significant (P = 0.003) non-linear relationship is noticeable: the largest SAI QRST was observed during the first 3 months after MI. At the same time, a positive correlation was observed after 3 months in the post MI period.

### Survival Analysis: Predictive Value of SAI QRST

**Figure 3:**
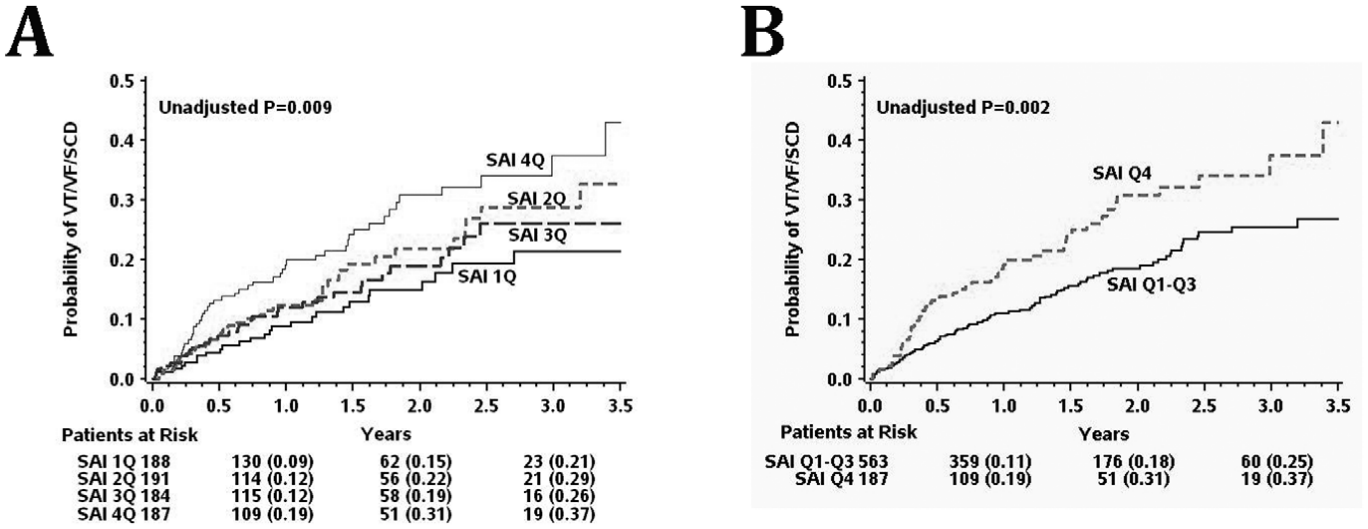
Kaplan-Meier curves for the probability of sudden arrhythmic death, (A) by quartiles of SAI QRST; (B) in patients with the highest SAI QRST quartile and those with the lower 3 SAI QRST quartiles.

**Figure 4:**
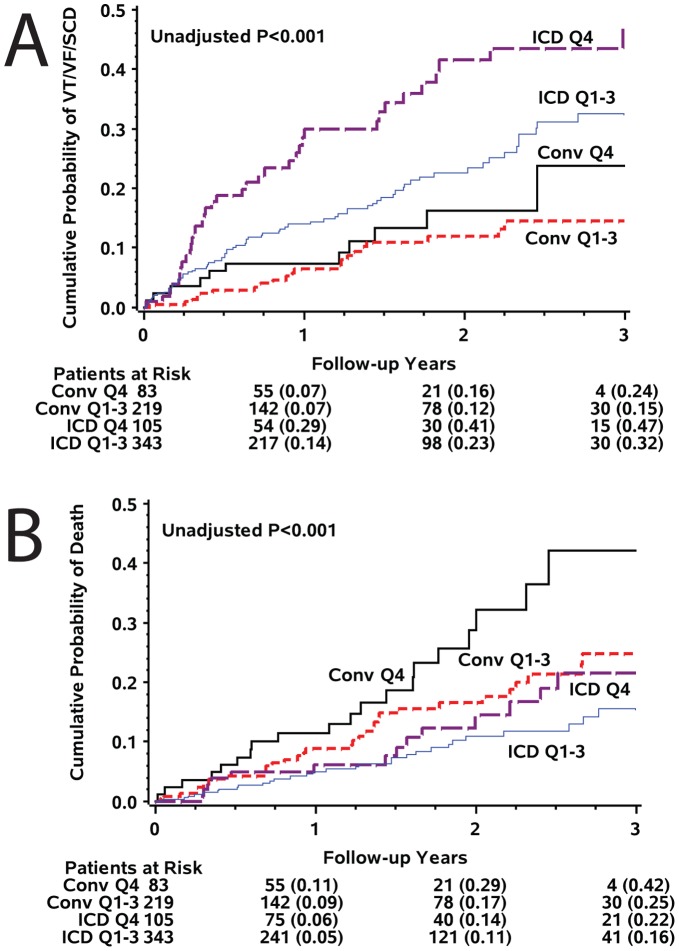
Kaplan-Meier curves for the probability of (A) sudden arrhythmic death, and (B) all-cause mortality in patients with the highest SAI QRST quartile and those with the lower 3 SAI QRST quartiles, by MADIT II study arm (ICD arm and conventional medical therapy arm).

Kaplan-Meier analysis showed statistically significant “dose-dependent” pattern of relationship between SAI QRST and SCD/VT/VF (Figure 3A): the larger SAI QRST the higher the risk. Probability of VT/VF/SCD was about twice higher amongst patients with SAI QRST in the highest quartile as compared to patients with SAI QRST in the 3 lower quartiles (Figure 3B). Importantly, large SAI QRST was associated with high risk of ventricular arrhythmia in both the ICD arm and in the conventional medical therapy arm (Figure 4A), although its predictive value was especially remarkable in the ICD arm, due to more frequent VT/VF events treated by ICD as compared to SCD cases in conventional medical therapy arm (Table 2). However, prediction of all-cause mortality by SAI QRST was more noticeable in the conventional medical therapy arm, rather than in the ICD arm (Figure 4B). Kaplan-Meier analysis survival curves showed a time-dependent pattern of all-cause mortality, which increased after 18 months of follow-up and possibly reflected increased non-sudden cardiac death due to heart failure progression, rather than complications of repeated acute coronary events (Table 2). Interestingly, SAI QRST seemed predictive for non-sudden cardiac death in both ICD arm and conventional medical therapy arm (Figure 4B).

In the multivariable Cox proportional-hazards regression model, after adjustment for other predictors of MADIT II study outcomes (age, BUN, QRS duration, atrial fibrillation, NYHA functional class), SAI QRST was an independent predictor of SCD/VT/VF and all-cause death (Table 3). Multivariable Cox model showed that increase in SAI QRST by 100 mV*ms would result in a 33% increase in the risk of SCD/VT/VF and a 27% increase in the risk of all-cause death.

**Table 3:** Hazard ratios for MADIT II risk score components for sudden arrhythmic death and all-cause mortality.

		SCD/VT/VF		Total mortality	
	Predictor	Hazard ratio (95% CI)	P value	Hazard ratio (95% CI)	P value
Without SAI QRST	NYHA CHF class >II	1.58(1.12–2.23)	**0.009**	1.67 (1.11–2.52)	**0.014**
	Atrial fibrillation	3.43(0.84–14.05)	0.087	3.26(0.45–23.72)	0.244
	QRS >120 ms	1.40(0.99–1.98)	0.060	1.61(1.07–2.41)	**0.023**
	Age >70yrs	1.07(0.73–1.57)	0.736	2.10(1.38–3.18)	**0.001**
	BUN >25 mg/dL	1.29(0.88–1.89)	0.186	1.98(1.30–3.02)	**0.002**
With SAI QRST	NYHA CHF class >II	1.57(1.11–2.21)	**0.011**	1.22(0.75–1.98)	0.419
	Atrial fibrillation	3.47(0.85–14.26)	0.084	3.42(0.47–24.99)	0.225
	QRS >120 ms	1.01(0.66–1.53)	0.977	2.00(1.31–3.06)	**0.001**
	Age >70y	1.00(0.67–1.47)	0.92	1.98(1.30–3.02)	**0.002**
	BUN >25 mg/dL	1.28(0.87–1.87)	0.212	1.66(1.10–2.51)	**0.015**
	SAI QRST per 100 mV*ms	1.33(1.11–1.59)	**0.002**	1.27(1.03–1.55)	**0.022**

### SAI QRST and Bundle Branch Block

**Figure 5:**
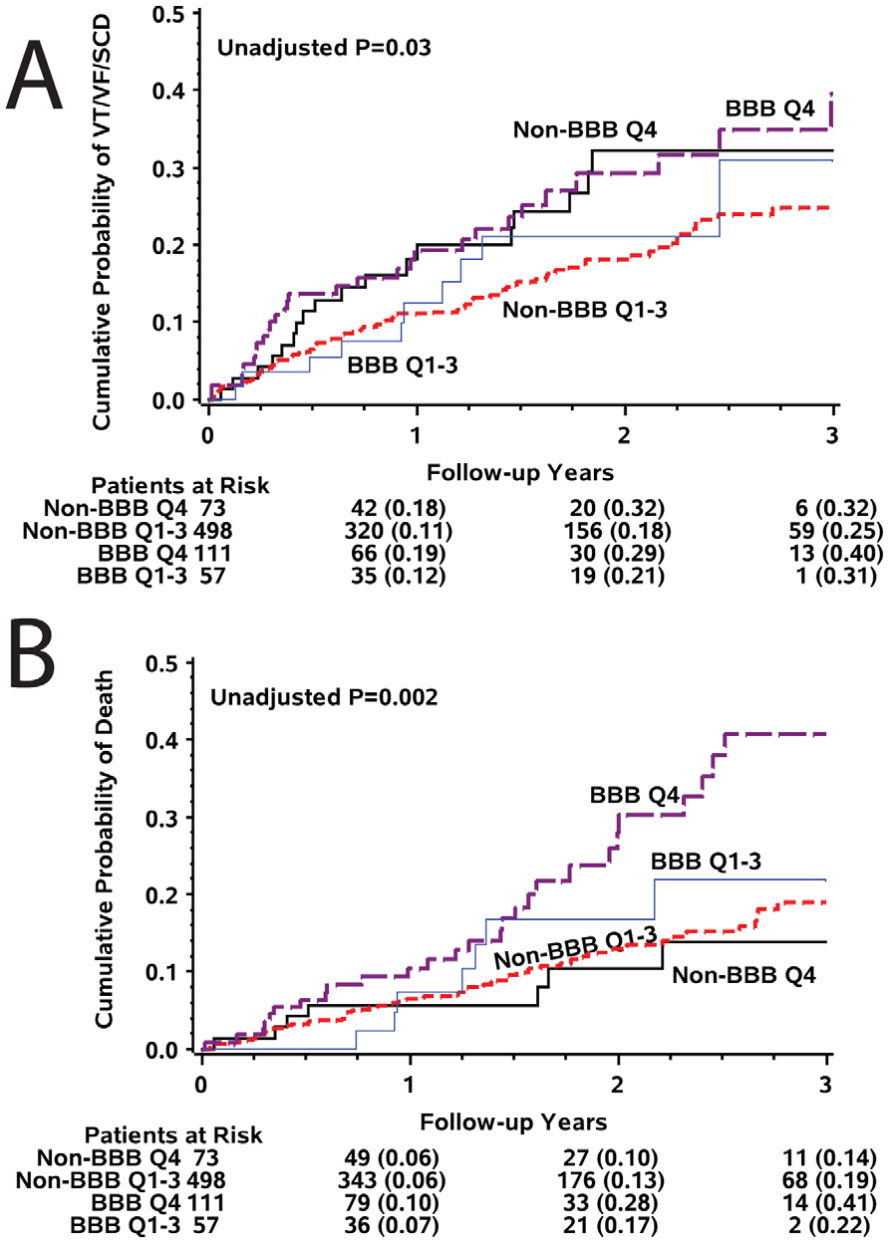
Kaplan-Meier curves for the probability of (A) sudden arrhythmic death, and (B) all-cause mortality in patients with the highest SAI QRST quartile and those with the lower 3 SAI QRST quartiles, by the presence of bundle brunch block.

No interaction between SAI QRST and BBB status was observed in multivariable Cox regression model (p = 0.987), and SAI QRST predicted SCD/VF/VF (Figure 5A) and all-cause mortality (Figure 5B) in patients with and without BBB. Interestingly, in the subgroups analysis SAI QRST predicted SCD/VT/VF in BBB patients, but all-cause death (predominantly non-sudden cardiac death) in non-BBB patients (Table 4).

**Table 4:** Multivariate Cox regression models for prediction of sudden arrhythmic death and all-cause mortality with SAI QRST as continuous variable in patients with and without bundle branch block.

			SCD/VF/VF		Total mortality	
	Predictor		Hazard ratio (95% CI)	P value	Hazard ratio (95% CI)	P value
All patients	BUN, mg/dL		1.02(1.00–1.03)	**0.042**	1.04(1.02–1.05)	**<0.001**
	CHF NYHA class>II		1.61(1.10–2.34)	**0.013**	1.12(0.73–1.71)	0.604
	SAI QRST per 100 mV*ms		1.30(1.09–1.56)	**0.004**	1.25(1.02–1.52)	**0.029**
	Bundle branch block		0.95(0.60–1.49)	0.808	1.40(0.85–2.31)	0.184
BBB patients	BUN, mg/dL	1.01(1.00–1.03)		**0.049**	1.04(1.02–1.05)	**<0.001**
	CHF NYHA class>II	1.62(1.11–2.36)		**0.012**	1.13(0.74–1.74)	0.573
	SAI QRST per 100 mV*ms	1.30(1.04–1.63)		**0.020**	1.17(0.78–1.73)	0.450
Non-BBB	BUN, mg/dL	1.01(1.00–1.03)		**0.049**	1.04(1.02–1.05)	**<0.001**
	CHF NYHA class>II	1.62(1.11–2.36)		**0.012**	1.13(0.74–1.74)	0.573
	SAI QRST per 100 pmV*ms	1.31(0.96–1.77)		0.088	1.28(1.02–1.61)	**0.035**

## Discussion

Our study showed that SAI QRST quantifies adverse electrical remodeling post-MI, which is characterized by wide QRS, increased magnitudes of spatial QRS and spatial T vectors, J-point deviation, and QT prolongation. We found that in MADIT II post-MI patients with and without BBB, increased SAI QRST independently predicted both VT/VF/SCD and all-cause death, in both the ICD arm and the conventional medical therapy arm. Enlarged SAI QRST was associated with increased risk after adjustment for all composites of the MADIT II risk score (age >70 y, BUN>25 mg/dL, QRS duration >120 ms, NYHA HF class>II, atrial fibrillation). Importantly, increased SAI QRST predicted outcomes in patients with and without BBB. This study showed that enlarged SAI QRST was associated with increased risk of sustained VT/VF, as well as non-sudden cardiac death and HF hospitalizations, but not hospitalizations due to recurrent MI (Table 2). Thus, the major finding of our study is that adverse electrical remodeling as quantified by increased SAI QRST carries independent risk of ventricular tachyarrhythmias, HF progression, and non-sudden cardiac death in post-MI patients with systolic dysfunction.

### SAI QRST as a Marker of Adverse Electrical Remodeling Post-MI

We proposed SAI QRST as a metric, which quantifies post-MI electrical remodeling. Previous experimental and clinical studies showed development of structural remodeling post-MI [6], characterized by dilatation and distortion of LV cavity shape, apoptosis, thinning, fibrosis and hypertrophy of remote from infarction sites [6], [7], scar formation and gradual remodeling of the scar [4] in the central and border zone areas [5], dilatation of LV cavity and/or other heart chambers, activation of the neurohumoral pathways, and left ventricular (LV) systolic and diastolic dysfunction. Larger infarcts tend to show greater increases in cell size in the non-infarcted areas, compared to smaller infarcts [7]. Development of regional hypertrophy post-MI is associated with enhanced regional heterogeneity of repolarization and steeper local gradient in spatial dispersion of repolarization, which is a well-known substrate vulnerable to ventricular arrhythmias. Complex structural and electrical remodeling post-MI result in a slowing and fractionation of ventricular conduction, dispersion of repolarization, occurrence of delayed afterdepolarizations [26] and create conditions for reentry and arrhythmogenesis [27]. LV hypertrophy is a strong, independent predictor of future cardiovascular events and SCD [28]–[30].

In addition, hibernating myocardium is characterized by regional myocyte hypertrophy as well [31]. Altered calcium uptake in the sarcoplasmic reticulum, inhomogeneity in sympathetic activation, and increase in interstitial connective tissue might lead to extremely high risk of SCD [31]. Despite significant functional improvement of hibernating myocardium after revascularization, inhomogeneity in myocardial sympathetic innervations might persist [32]. Thus, increased SAI QRST might be a marker of hibernating myocardium or residual structural remodeling, persisting after revascularization. Further studies are needed to test this hypothesis.

Importantly, as the MADIT II study included post-MI patients only, results of this study should not be simply extrapolated on other populations. Further studies are needed to answer the question if SAI QRST is predicting electrical remodeling in the ventricles with different underlying pathophysiology.

Ultimately post-MI remodeling of cardiac structure is characterized [33] by changes in (1) cell geometry (size and shape), (2) gap junctions (distribution and conductivity), and (3) interstitial space (size and distribution). These changes could result in increased curvature of the wavefront [34] and significant regional slowing of conduction. Slowing of conduction is a fundamental condition for reentrant circuit: the lower the conduction velocity, the smaller the area in which a reentrant circuit can occur [35].

In our study, the largest SAI QRST was observed in patients with recent MIs (<3 month, Figure 2), when hypertrophy had not been developed yet. We speculate that the Brody effect is responsible for significantly enlarged SAI QRST in early (<3 months) post-MI adverse remodeling. The Brody effect explains that heart dipoles oriented perpendicular to the assumed spherical border zone between high- and low- conductance layers are effectively enhanced, resulting in larger surface potentials [36]. On the other hand, those dipoles tangential to the high-low conductivity tissue border are effectively diminished, resulting in lowered surface potentials [37]. Progressive enlargement of the infarcted ventricle and thinning and distension of the LV cavity can result in a near-spherical shape of LV and elicit the Brody effect on the blood-tissue border. Future theoretical studies are needed to prove this hypothesis.

### Correlation between the Time after Myocardial Infarction and SAI QRST

Obviously, multiple confounding factors (age of patient, size of the scar, preexisting LVH and other significant factors, listed in Table 1) affect the significant nonlinear relationship between the time after MI and SAI QRST. However, all these factors taken together helped to reconcile SAI QRST as possibly being a time-dependent marker of post-MI electrical remodeling. Prospective longitudinal study is needed to prove this hypothesis.

### Contradictory Findings in SAI QRST

Surprisingly, in our previous preliminary analysis of an ongoing prospective study of patients with primary prevention ICD diminished, but not increased SAI QRST was associated with increased risk of VT/VF with appropriate ICD therapies [17], [18]. Our data were obtained in somewhat different patients populations. In our earlier study patients with both ischemic and non-ischemic cardiomyopathy were enrolled, whereas MADIT II enrolled post-MI patients only. More females and African-Americans were included in our prior study. Differences with current data can be ascribed to these differences in studied populations, but importantly, SAI QRST was a strong, independent predictor of outcomes in both, independently performed, studies. We currently can only speculate on the underlying mechanism. One possible explanation came from modeling studies, which showed that LV hypertrophy could be presented by both diminished and increased magnitudes of spatial QRS and T vector [38], [39]. It is possible that different underlying pathophysiology might result in a different pattern of SAI QRST changes. Specifically, differences in the activation distribution and in the heart and body surface geometry can be due to underlying pathophysiology. This hypothesis is indirectly supported by the experimental studies, which identified signaling pathways, affecting some, but not all features of remodeling in different disease models [16], [40]. Further theoretical and clinical studies are needed to clarify this intriguing discrepancy. Future large population studies might help to detect U-shaped risk associated with SAI QRST.

### Risk Stratification in Post-MI Patients with BBB

Analysis of ECG in BBB is challenging and frequently inconclusive. Therefore, our finding of strong, independent predictive value of SAI QRST in BBB patients is clinically important and promising. In this study, SAI QRST predicted outcomes after adjustment for BBB status and QRS duration. Interestingly, in BBB patients, increased SAI QRST more strongly predicted sustained VT/VF events with appropriate ICD therapies, rather than mortality. At the same time in patients without BBB, SAI QRST predicted mortality rather than appropriate ICD therapies.

### ECG Predictors of Outcomes in MADIT II

Multiple ECG markers of SCD risk have been tested in MADIT II data analysis, but only a few demonstrated independent predictive value after adjustment for clinical predictors of outcomes. Increased repolarization lability (QT variability and T wave variability) has been shown to be significant, independent predictor of appropriate ICD therapies in the ICD arm [21], [41], [42], but did not predict SCD in the conventional medical therapy arm. Frequent ventricular premature beats (>3/10 min) were associated with death in the conventional medical therapy arm and with appropriate ICD therapy in the ICD arm [43], whereas heart rate turbulence was not predictive. In post-MI patients with resolved Q waves, fragmented QRS was associated with increased risk of cardiac events [44], but was not associated with cardiac death independently of Q waves. Prolonged QRS duration predicted SCD in the conventional medical therapy arm [45], but did not predict VT/VF/SCD in the ICD arm. Thus far, SAI QRST seems to be the only ECG predictor of both VT/VF/SCD and total mortality in both MADIT II arms, which underscores the importance of post-MI adverse electrical remodeling in the pathogenesis of arrhythmia and heart failure progression.

### Limitations

This was a retrospective analysis of prospectively collected data of randomized controlled trial MADIT II, and, therefore, statistical power of subgroups analysis was limited. Patients without an available ECG have been excluded from this analysis. However, absence of statistically significant interaction between SAI QRST and BBB status in the Cox model suggests a similar effect of SAI QRST on survival in patients with and without BBB. Validation of predictive value of SAI QRST in a prospective study is needed before its implementation into clinical practice.

As previously shown, appropriate ICD therapies may overestimate the frequency of SCD [45]. However, study of the predictors of ventricular arrhythmia is critical for understanding SCD mechanisms and developing mechanistically- sound risk markers of SCD.

As MADIT II study population is predominantly presented by white males, we did not apply race- and gender-specific thresholds for SAI QRST in this study. At the same time, voltage ECG parameters are known to be gender-specific [46] and further development of gender- and possibly race-specific thresholds of SAI QRST will be needed in the future.

SAI QRST is significantly different in patients with BBB as compared to those without BBB [18]. Therefore, threshold of SAI QRST that is specific for ventricular conduction status might demonstrate better precision in prediction of outcomes. However, in this study we utilized one uniform threshold of SAI QRST for patients with and without BBB to simplify its implementation.

In this study only baseline ECGs were analyzed. Longitudinal changes in SAI QRST over post-MI time course in correlation with longitudinal changes in LV morphology, assessed by imaging, are unknown. Future theoretical and prospective longitudinal clinical studies are needed to confirm whether or not SAI QRST is indeed a marker of electrical remodeling.

### Conclusions

Adverse electrical remodeling as quantified by increased SAI QRST carries independent risk of ventricular tachyarrhythmias, HF progression and non-sudden cardiac death in post-MI patients with systolic dysfunction. In post-MI MADIT II patients with and without BBB, increased SAI QRST independently predicted both VT/VF/SCD and all-cause death, both in the ICD arm and in the conventional medical therapy arm. Enlarged SAI QRST was associated with increased risk after adjustment for all composites of the well-known MADIT II risk score (age >70 y, BUN>25 mg/dL, QRS duration >120 ms, NYHA HF class>II, atrial fibrillation).

### Clinical Implications

The development of our novel ECG marker of adverse electrical remodeling, SAI QRST, and demonstration of its predictive value for SCD/VT/VF and all-cause mortality in a blinded analysis of landmark ICD trial MADIT II opens up new opportunities for SCD risk stratification. Future studies of SAI QRST are warranted and might uncover novel mechanisms of electrical remodeling in the post-infarction period.
